# Imaging of supratentorial intraventricular masses in children:a pictorial review— part 1

**DOI:** 10.1007/s00234-024-03314-1

**Published:** 2024-03-11

**Authors:** Fabrício Guimarães Gonçalves, Mario E. Mahecha-Carvajal, Aishwary Desa, Harun Yildiz, Jawabreh Kassem Talbeya, Luz Angela Moreno, Angela N. Viaene, Arastoo Vossough

**Affiliations:** 1 Radiology Department, Children’s Hospital of Philadelphia, Philadelphia, USA; 2 University of the Andes, Cra. 1 #18a-12, Bogotá, Colombia; 3 Drexel University College of Medicine Philadelphia, Philadelphia, PA USA; 4 Department of Radiology, Dortcelik Children’s Hospital, Bursa, Turkey; 5 Bnai-Zion Medical Center, Haifa, Israel; 6 Pediatric Imaging, Department of Radiology, Fundación Hospital La Misericordia, Universidad Nacional de Colombia, Bogotá, Colombia; 7 Perelman School of Medicine, University of Pennsylvania, Philadelphia, USA; 8 Radiology Department, Children’s of Alabama, Alabama, USA; 9 Pathology Department, Children’s Hospital of Philadelphia, Philadelphia, USA

**Keywords:** Supratentorial intraventricular masses, Choroid plexus tumours, Magnetic resonance imaging, World Health Organization Classification of Tumours

## Abstract

**Purpose:**

This article is the first in a two-part series designed to provide a comprehensive overview of the range of supratentorial intraventricular masses observed in children. Our primary objective is to discuss the diverse types of intraventricular masses that originate not only from cells within the choroid plexus but also from other sources.

**Methods:**

In this article, we review relevant epidemiological data, the current genetics/molecular classification as outlined in the fifth edition of the World Health Organization’s Classification of tumours of the Central Nervous System and noteworthy imaging findings. We conduct an exhaustive analysis of primary choroid plexus tumours as well as other conditions such as choroid plexus hyperplasia, choroid plexus cyst, choroid plexus xanthogranuloma, atypical teratoid rhabdoid tumour, meningioma, arteriovenous malformation and metastasis.

**Results:**

We comprehensively evaluated each supratentorial intraventricular mass, providing an in-depth analysis of their unique clinical and histological characteristics. The fifth edition of the World Health Organization Classification of Tumours of the Central Nervous System introduces major modifications. These important changes could potentially have a profound impact on the management strategies and subsequent outcomes of these tumours.

**Conclusion:**

Intraventricular masses in children can arise from various sources. Surgical intervention is key for certain supratentorial intraventricular masses in paediatric patients, with preoperative neuroimaging essential to decide the best treatment approach, surgical or otherwise, as some cases may not require surgery.

## Introduction

In children, a variety of masses (both benign and malignant) can occur within or adjacent to the supratentorial intraventricular compartment. These lesions exhibit a wide range of histological characteristics and can originate from several types of cells, including those within the choroid plexus (CP), those forming the ventricular lining, those residing within the septum pellucidum and brain parenchyma cells, with some masses projecting into the ventricles (Table 1) [1].

**Table 1:** Supratentorial intraventricular masses based on their cells of origin

Choroid plexus	Ventricular lining	Septum pellucidum	Brain parenchyma
Primary choroid plexus tumours Choroid plexus hyperplasia Choroid plexus cyst Choroid plexus xanthogranuloma Choroid plexus lipoma ATRT Meningioma AVM Teratoma Cavernous malformation Metastasis	Colloid cyst SEGA Ependymoma Ganglioglioma Myxoid glioneuronal tumour ATRT AVM	Central neurocytoma High-grade glioma Pilocytic astrocytoma ATRT Cavernous malformation Myxoid glioneuronal tumour Other embryonal tumours AVM	High-grade glioma Pilocytic astrocytoma Ganglioglioma ATRT Cavernous malformation Other embryonal tumours

*ATRT*, atypical teratoid rhabdoid tumour; *AVM*, arteriovenous malformation; *SEGA*, subependymal giant cell astrocytoma

Symptoms presented by supratentorial intraventricular masses can vary and often overlap among conditions. Patients generally exhibit signs and symptoms of increased intracranial pressure, typically attributed to the overproduction of cerebrospinal fluid (CSF) or obstruction of CSF outflow, resulting in hydrocephalus [2]. Common features include macrocephaly, widened sutures, bulging fontanelles, altered mental status, headache, hydrocephalus, papilledema, nausea, vomiting, cranial nerve deficits, diplopia, ataxia and seizures [3–5]. Less commonly observed features include subarachnoid, intraventricular and/or intratumoral haemorrhage, focal neurologic deficits and in rare instances, psychosis or bobblehead doll syndrome [2]. Indolent and benign lesions may be discovered incidentally or may resolve spontaneously, leaving patients asymptomatic.

Moreover, masses occurring within the supratentorial intraventricular system may present overlapping imaging features in children. Therefore, a biopsy for tissue diagnosis is typically necessary for accurate treatment planning. Furthermore, resection without adjuvant therapy may be curative in selected cases [6]. Given the complexity of supratentorial intraventricular masses in paediatric patients, detailed preoperative neuroimaging is crucial for surgery planning. Understanding the typical imaging characteristics aids in establishing a diagnosis, especially when tissue samples are unavailable. This is vital since a subset of these lesions may be managed expectantly without immediate surgical intervention [6].

This article is the first of a two-part series on supratentorial intraventricular masses in children. The aim of this paper is to review several common and rare causes of masses arising in the supratentorial ventricular system and propose a method to classify them preoperatively.

In part 1, we will review lesions that arise from cells within the CPs. It is important to note that lesions originating from the pineal gland and pineal recess, sellar and suprasellar region and prepontine cistern may eventually extend into the third ventricle and manifest as an “intraventricular mass”. However, these lesions are beyond the scope of this review.

## Approach to differential diagnosis

We provide an approach to assist with narrowing the differential diagnosis of primary intraventricular masses in children. The first step is to decide if the lesion is purely intraventricular, intraventricular with parenchymal extension or extra-ventricular origin with intraventricular extension or uncertain. The latter category will correspond to a very small percentage of cases and are beyond the scope of this review.

**Fig. 1:**
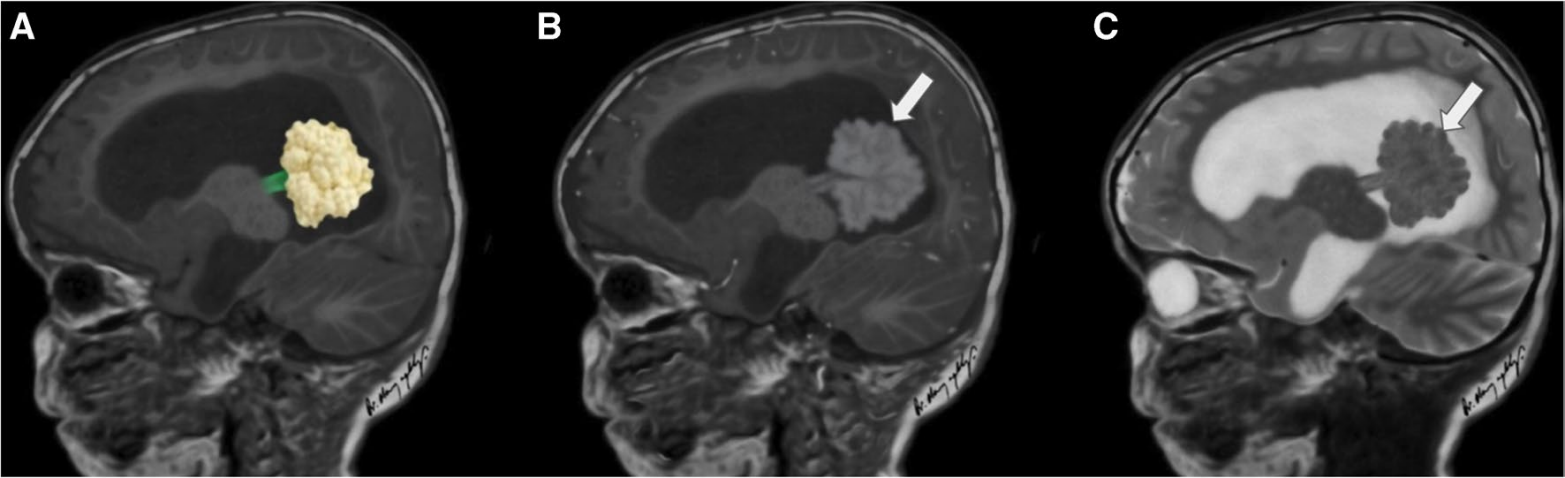
Three illustrations (**A**, **B**, **C**) depict the typical imaging features of a choroid plexus papilloma (white arrows). A choroid plexus papilloma is typically a mass with a frond-like or cauliflower-like appearance, surrounded by cerebrospinal fluid and there is no invasion of the adjacent brain parenchyma

**Fig. 2:**
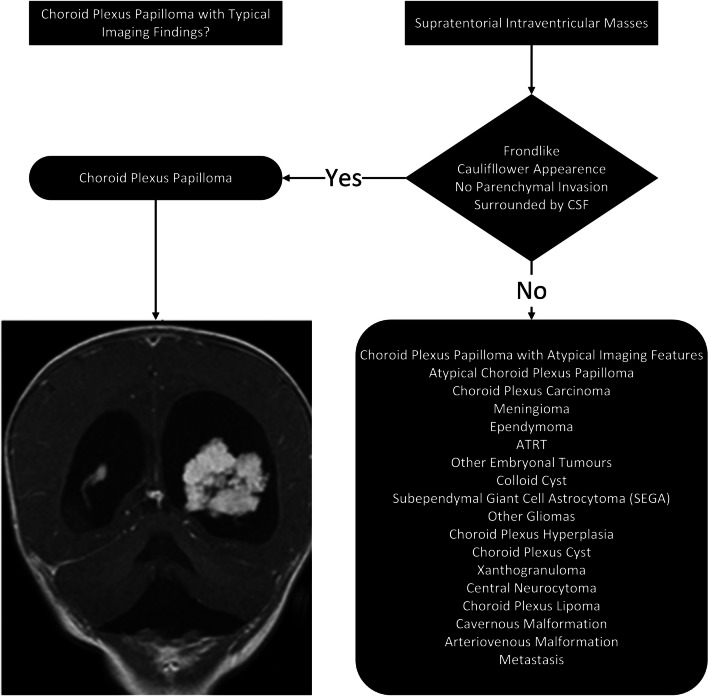
The second step to preoperatively classify supratentorial intraventricular tumours in children involves looking for a frond-like mass typically with no parenchymal invasion and that is surrounded by cerebrospinal fluid, which is typical for a choroid plexus papilloma. It is important to remember that a number of choroid plexus papilloma may also show some degree of parenchymal invasion

Since the most common intraventricular mass in children is a CP papilloma (CPP) [7] and the majority of CPPs demonstrate typical imaging findings the second step is to assess if the intraventricular mass is a CPP. Intraventricular CPPs typically demonstrate a characteristic imaging pattern with a frond-like or cauliflower appearance, surrounded by CSF with no evident invasion of the brain parenchyma (Fig. 1). However, several intraventricular tumours do not have a cauliflower appearance, including CPPs with atypical neuroimaging features (Fig. 2).

**Fig. 3:**
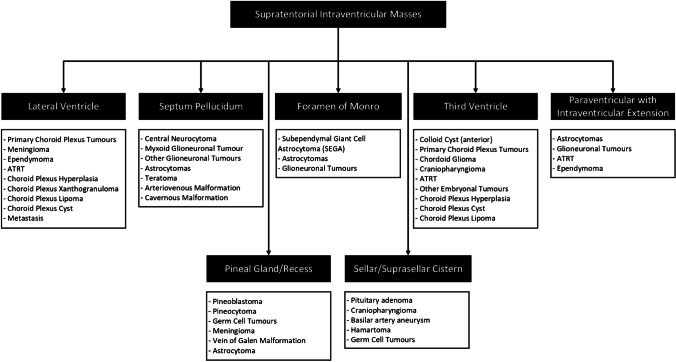
The third step to preoperatively classify supratentorial intraventricular masses in children is based on location. The flowchart classifies various intraventricular masses based on their typical location. Metastases are more common in the choroid plexus, but can occur in multiple locations and are typically multiple in numbers. Masses in the pineal gland/recess and sellar/suprasellar cistern with intraventricular extension are not within the scope of this review

A third step involves narrowing the differential diagnosis based on the location of supratentorial intraventricular masses. These masses, depending on their location, can be further classified into lesions arising from the septum pellucidum, lateral ventricles, foramen of Monro, third ventricle or paraventricular structures (Fig. 3). Pineal gland/recess and sellar/suprasellar cistern masses may also show intraventricular extension; however, these are not within the scope of this review.

**Fig. 4:**
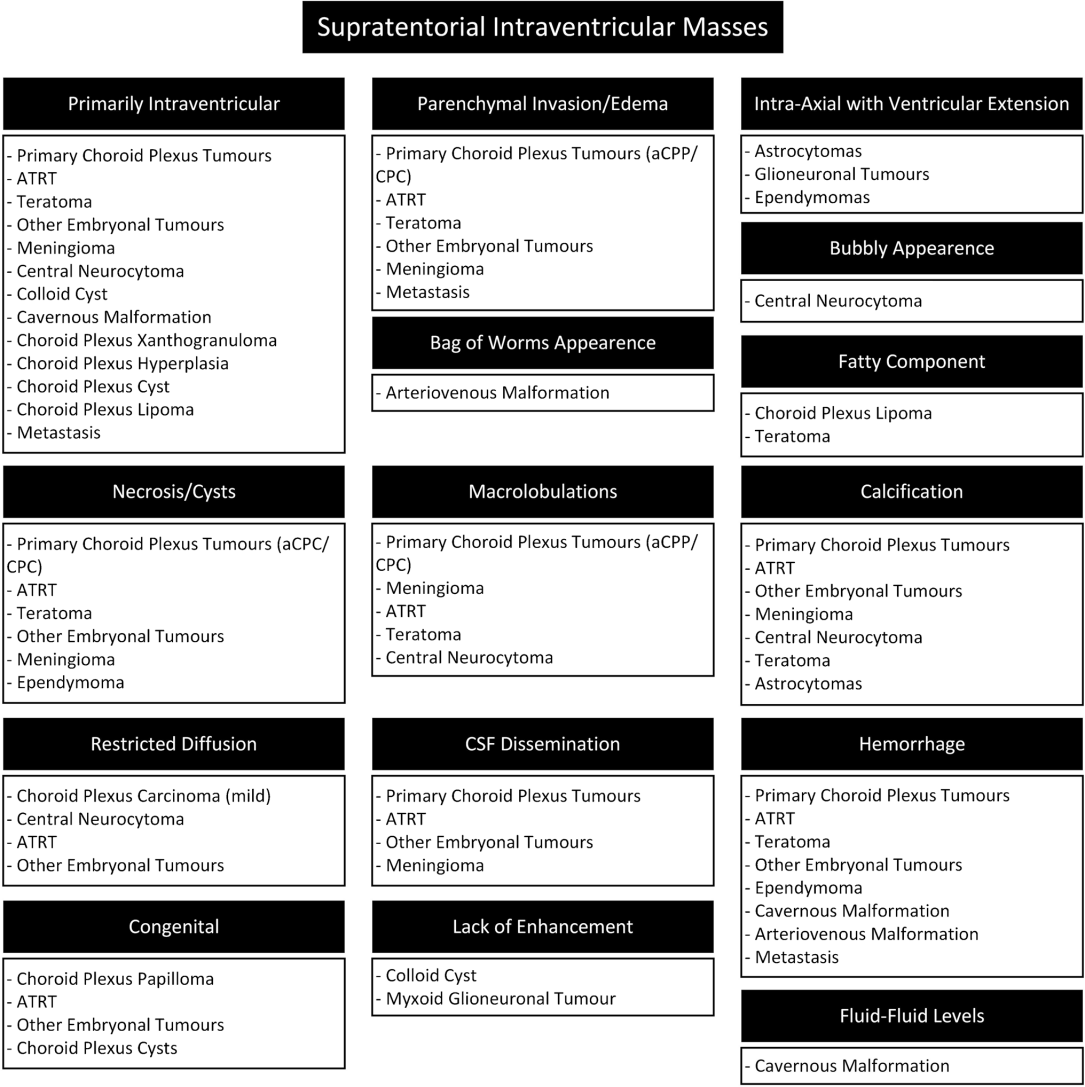
The fourth step to preoperatively classifying supratentorial intraventricular tumours in children, based on additional imaging features

Finally, a fourth step is to refine the differential diagnosis based on the specific imaging features and components of these masses. Additional features that can assist in the differential diagnosis are age at presentation, an intraventricular location as the primary origin, ventricular extension of intra-axial lesions, intraventricular lesion with parenchyma invasion, CSF dissemination, lack of enhancement, necrosis and/or large cysts, bubbly appearance, macrolobulation, restricted diffusion, fluid–fluid level, fatty components, CSF signal intensity or “bag of worms” appearance (Fig. 4).

## Features of supratentorial intraventricular masses during foetal life

There is a scarcity of comprehensive neuroimaging research on antenatal imaging of congenital brain tumours (CBTs), particularly those located in the ventricular system. Collaborative multi-institutional research would foster a comprehensive understanding of the origins, biological behaviour, optimal diagnostic approaches and ideal treatment strategies for these rare ventricular masses during foetal life.

CBTs have an approximate occurrence rate of 0.34 per 1,000,000 live births, representing only 10% of all antenatal tumours [8], typically diagnosed incidentally during the second or third trimester [9]. Among CBTs, teratomas are the most prevalent, accounting for at least half of the cases [10]. However, due to their large and heterogeneous appearance on prenatal imaging, distinguishing between an intraventricular location and ventricular compression can be challenging. The complex composition of teratomas, comprising solid and cystic components, often obscures precise anatomical boundaries within the ventricular system. The potential for teratomas to attain massive sizes and cause significant clinical implications emphasises the need for further research and collaborative efforts to comprehend their pathogenesis and optimise diagnostic and treatment strategies [10].

Other supratentorial intraventricular tumours can occur in the ventricular system such as high grade gliomas (HGGs), subependymal giant cell astrocytomas (SEGAs), CPPs, atypical teratoid rhabdoid tumours (ATRTs) and ependymomas HGGs are the most common congenital astrocytic tumour, typically manifesting as a large supratentorial mass leading to midline structure displacement, obstructive hydrocephalus and an augmented head circumference. These tumours are detected towards the conclusion of gestation when compared to other CBTs, specifically during the end of the third trimester [11]. Congenital SEGAs are often suspected when multiple cardiac rhabdomyomas are identified during prenatal ultrasound examinations at the location of the foramen of Monro. The survival rate is approximately 71% overall, accompanied by an elevated incidence of foetal mortality [10, 12].

Choroid plexus tumours (CPTs) typically exhibit a frond-like morphology and exhibit abundant vascularity alongside ventriculomegaly. Prenatal imaging does not provide reliable discriminatory features to differentiate CPP from choroid plexus carcinoma (CPC). Additionally, choroid plexus CP hemorrhage can mask an underlying CPT, requiring postnatal confirmation [10, 13]. ATRTs commonly manifest as large heterogeneous masses with solid, cystic and necrotic components that are located in different compartments, including supratentorial, infratentorial, intraventricular or suprasellar regions [10]. Ependymomas may manifest as large multilobulated lesions located either in the supratentorial or infratentorial regions, featuring intrinsic calcifications. These lesions may extend into the posterior fossa and even into the spinal canal [14].

## Choroid plexus

The CP constitutes a complex epithelial-endothelial convolute composed of epithelium, stroma and rich vascular supply. The stroma houses fibroblasts, inflammatory cells and a dense extracellular matrix [15]. There are four primary clusters of CP, each occupying either the lateral, third or fourth ventricles.

The CP is the primary source of CSF and it has been implicated in autoimmune inflammation within the central nervous system (CNS) through the expression of major histocompatibility complex classes I and II on CP epithelial cells [15]. The CP may play a role in a broad spectrum of congenital or acquired CNS pathologies, either as a primary organ (including neoplasms) or through disease extension. Although imaging patterns of these disorders are not always diagnostic, they are instrumental in highlighting the abnormality and narrowing the differential diagnosis. Supplementary clinical information, CSF studies and biopsy may be necessary to make a definitive diagnosis [16].

Primary CPTs originate from neuroectodermal cells and range from grade 1 CPPs to grade 3 CP carcinomas (CPCs). It remains unclear whether diffuse villous hyperplasia of the CP, a rare congenital condition, represents a precursor lesion deserving inclusion in this disease spectrum [17].

### Choroid plexus papilloma

### Background

CPPs are benign intraventricular neuroepithelial tumours that primarily originate from the CP. These lesions can be detected at any age but are more commonly identified during the first year of life, with a median age of 3.5 years. CPPs are rare, representing only 0.4 to 0.8% of all intracranial tumours [7]. In children, they are more frequently found in the atrium of the lateral ventricles and less often in the third ventricle. Most CPPs arise within the ventricular system, with few instances of these tumours emerging within the brain parenchyma or disseminating throughout the neuraxis [2]. Their estimated average annual incidence is 0.3 cases per 1 million [7].

### Pathology

**Fig. 5:**
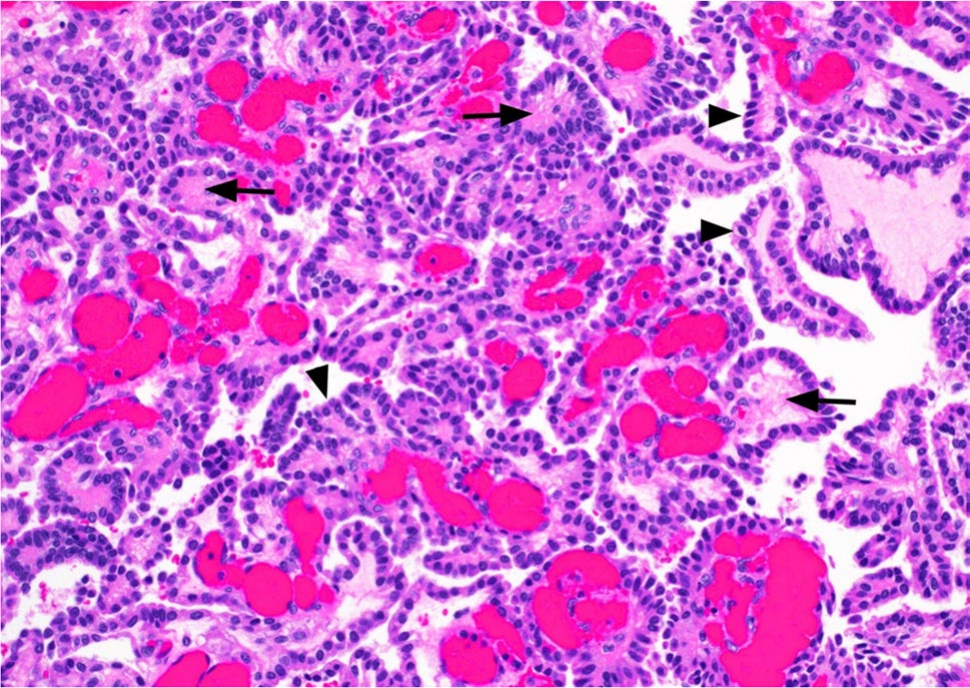
Choroid plexus papilloma—the image displays a tumour with a papillary architecture, composed of fibrovascular cores (indicated by arrows) that are lined by bland, cuboidal to columnar epithelium (indicated by arrowheads). H&E stain, 20 × magnification

Macroscopically, CPPs are well-circumscribed, soft, globular, often papillary, friable pink masses with irregular projections and high vascularity [18]. Microscopically, CPPs share similarities with the normal CP, consisting of papillary neoplasms composed of single or multiple layers of a cuboidal to columnar epithelium with a delicate fibrovascular core (Fig. 5) [18]. However, in contrast to the orderly cobblestone-like epithelium of the normal CP, CPPs are hypercellular and exhibit more cellular crowding and stratification.

According to the 2021 World Health Organization (WHO) Classification of Tumours of the CNS (WHO CNS5), CPPs are classified as grade 1, demonstrating fewer than two mitotic figures per 10 high-power fields. The diagnostic criteria include the demonstration of CP differentiation through histopathological and immunophenotypic features, absent or low mitotic activity and intraventricular or cerebellopontine angle location. Although adherence to ventricular walls may occur, brain invasion and necrosis are uncommon [18, 19].

CPPs are easily recognisable by their histology. Genome-wide chromosomal copy-number analysis can demonstrate characteristic hyperploidy [20]. Additionally, CPPs may also show typical epigenetic signatures [21].

### Neuroimaging features

CPPs typically appear as lobulated, intraventricular masses with a homogeneous enhancement, presenting a frond-like or cauliflower-like appearance. A variable degree of calcification can be seen in around 25% of cases, which is more commonly fine and speckled. Enlargement of the choroidal artery can be detected on both angiographic and cross-sectional imaging. Aggressive features such as irregular margins, invasion of the adjacent brain or surrounding oedema are uncommon [16]. Metastasis may be present at the time of diagnosis in approximately 17% of cases [22].

In cases of open fontanelles, neurosonography may play a role in evaluating CPPs. In neurosonography, CPPs appear as uniformly echogenic, lobulated intraventricular masses. These masses may display bidirectional flow throughout the diastole, indicative of blood flow through vessels arranged chaotically [23].

On CT scans, CPPs appear as well-defined, frond-like, isodense or slightly hyperdense lesions within the ventricles. They are typically associated with homogeneous enhancement and ventriculomegaly [24]. Calcifications may also be present.

**Fig. 6:**
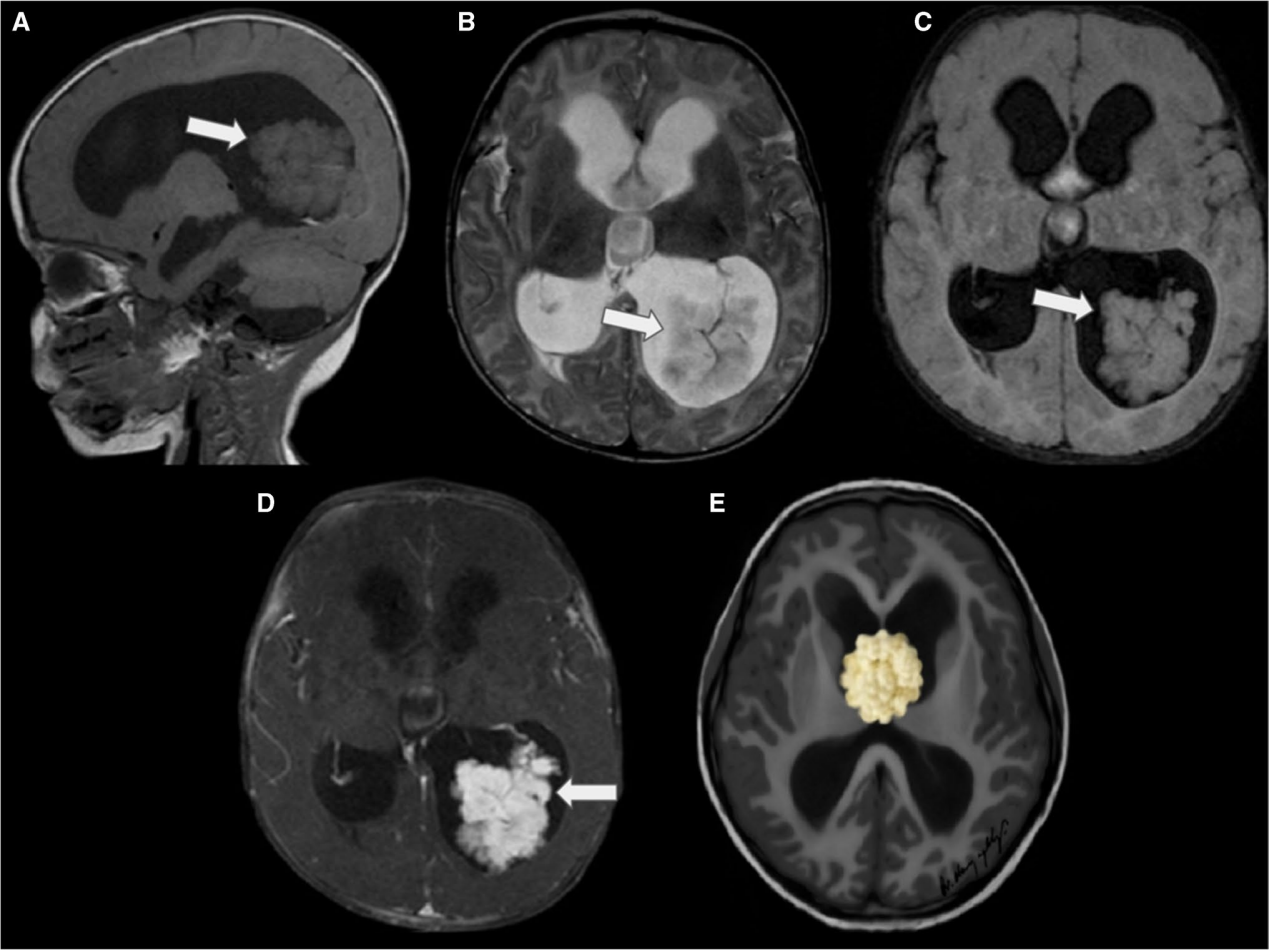
Choroid plexus papilloma in a 2-year-old boy with headaches, presenting the typical frond-like, lobulated and cauliflower-like features (white arrows). **A** A sagittal T1-weighted image shows a well-defined intraventricular mass with an isointense signal and moderate hydrocephalus. **B** An axial T2-weighted image reveals that the lesion is predominantly hyperintense, with central internal increased vascularity. **C** An axial FLAIR image indicates that the lesion is predominantly isointense, with no signs of invasion or oedema of the adjacent brain. **D** An axial contrast-enhanced T1-weighted image demonstrates marked enhancement of the lesion. In this particular case, the contrast-enhanced image emphasises the frond-like appearance. The microlobulated contours of the choroid plexus are evident, with CSF freely bathing among the papillary tumours. **E** An illustration depicts a third ventricular choroid plexus papilloma with hydrocephalus, but no signs of brain invasion

On MRI scans, CPPs are typically present as well-defined, intraventricular, frond-like lobulated masses, with an isointense to hypointense signal on T1WI and a hyperintense signal on T2WI. Variable degrees of flow voids may be observed, indicating active blood flow. Due to their rich vascularity, these tumours typically display moderate to strong enhancement, owing to their rich vascularity (Fig. 6) [16]. It has been observed that perfusion weighted imaging can differentiate CPPs from other etiologies with similar morphological characteristics, such as intraventricular meningioma and  CPCs. Among those techniques, dynamic susceptibility contrast provides maps of cerebral blood volume (CBV) and noninvasive measurements of relative cerebral blood volume (rCBV). Thus, the rCBV parameter correlates with tumour vascularity and is increased in tumours with a high rate of pathologic angiogenesis. In the case of CPPs, these demonstrate low rCBV values, whereas intraventricular meningioma and  CPCs present higher rCBV values [25, 26].

## Atypical choroid plexus papilloma

### Background

Atypical CPPs (aCPPs) were first classified in the 2007 WHO Classification of Tumours of the CNS to identify CPTs with intermediate histology between CPPs (WHO grade 1) and CPCs (WHO grade 3). These tumours are designated as WHO grade 2 and distinguished from CPPs by increased mitotic activity on histology, which is associated with a higher tumour recurrence after curative surgery [27]. According to the WHO CNS5, “an aCPP is a CPP that exhibits increased mitotic activity but does not fulfil the criteria for a CPC” [28].

### Pathology

Histologically, aCPPs are distinguished from CPPs based on increased mitotic activity (≥ 2 mitoses/10 high power fields, equating to a mitotic count of ≥ 1 mitosis/mm^2^). They may also exhibit increased cellularity and nuclear pleomorphism compared to CPPs. These histologic features in aCPPs have been found to be associated with a 4.9-fold higher recurrence after five years. In select cases, demonstrating hyperploidy through genome-wide chromosomal copy-number analysis may be desirable. Otherwise, aCPPs are identical to typical CPPs [29]. Genome-wide chromosomal copy-number analysis can reveal characteristic hyperploidy [20], which may be helpful in differentiating them diagnostically from CPCs.

### Neuroimaging features

It has been suggested that signal characteristics and enhancement patterns cannot differentiate between benign and malignant CPTs [30]. Lin et al. [31] have demonstrated that invasion and oedema of the brain parenchyma are signs of malignancy in CPTs, which can occasionally be observed in aCPPs. aCPPs tend to exhibit a lesser degree of papillary appearance and more commonly show a lobulated contour, typically enhancing after contrast injection [31].

On CT scans, aCPPs appear as isodense or slightly hyperdense with a lobulated or papillary shape. In addition, they exhibit intense contrast enhancement and hydrocephalus [32].

**Fig. 7:**
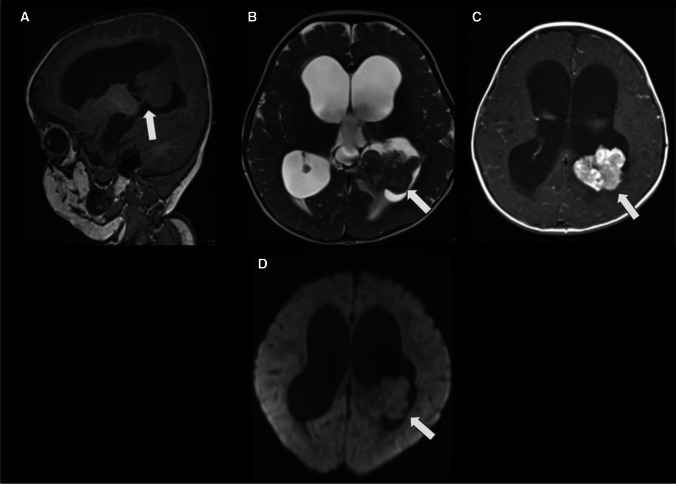
Atypical choroid plexus papilloma (whiter arrows) in an 18-month-old girl with headaches and recent seizures. **A** A sagittal T1-weighted image shows a lobulated, hypointense mass with suggestion of brain invasion and moderate hydrocephalus. **B** An axial T2-weighted image reveals that the lesion is predominantly hypointense, with peripheral increased vascularity. In addition, there are signs of brain invasion and a moderate degree of surrounding vasogenic oedema. **C** An axial contrast-enhanced T1-weighted image demonstrates marked and heterogeneous enhancement of the lesion. **D** An axial diffusion-weighted image indicates that the mass is isointense in comparison with the adjacent brain

On MRI, aCPPs may exhibit a papillary, lobulated or irregular appearance. Similar to other CPTs, they appear hypointense or isointense on T1WI and hyperintense or isointense on T2WI. Like grade 1 tumours, they show moderate or strong enhancement after contrast, but the internal signal is more heterogeneous, indicative of a greater incidence of calcification, haemorrhage and necrosis (Fig. 7) [31].

### Choroid plexus carcinoma

### Background

CPCs are the most aggressive neuroectodermal tumours, primarily originating from the CP. Although rare, CPCs are more aggressive than CPPs or aCPPs [33]. Approximately 80% of all CPCs are detected in children, with a median age of incidence of 3 years [34]. They constitute 15–20% of CPTs and are most commonly found in the lateral ventricles [34]. Progression from a CPP may occur in less than 20% of cases; however, most CPCs occur de novo [35]. Around 50% of CPC patients harbour TP53 mutations in somatic cells. There is a strong association between CPC and TP53 germline mutations. Furthermore, paediatric CPC cases frequently have TP53 germline mutations in association with Li-Fraumeni syndrome [36–38].

### Pathology

**Fig. 8:**
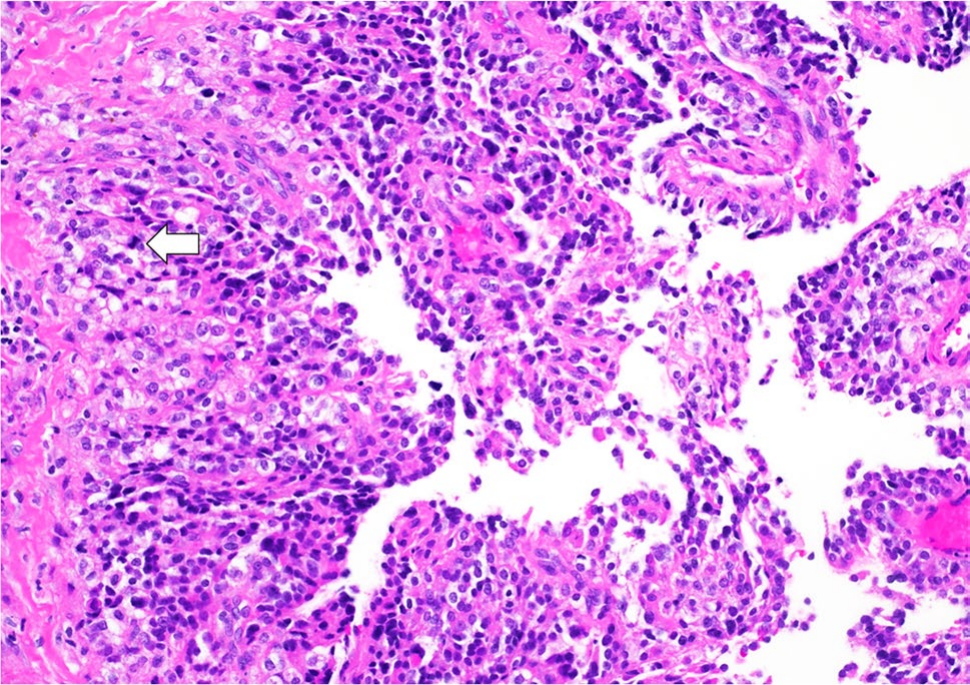
Choroid plexus carcinoma—the image displays a tumour with sheet-like growth, focal papillary architecture, large atypical cells and mitotic activity (indicated by the arrow). Foci of necrosis are present, but not shown in this image. H&E stain, 20 × magnification

Unlike CPPs, CPCs tend to present as invasive lesions, often with CSF dissemination or subarachnoid spread [33, 39]. Histologically, CPCs exhibit a less conspicuous papillary pattern, with tumour cells frequently arranged in dense sheets. Notable features of anaplasia, including nuclear atypia, cellular pleomorphism, increased mitotic activity and necrosis, are observed, and these may be focal or diffuse (Fig. 8). Brain invasion and necrosis are commonly seen [18, 19].

According to the WHO CNS5, CPPs are classified as grade 3. The diagnostic criteria include the demonstration of CP differentiation by histopathological and immunophenotypic features and the presence of at least four of the following five histological features: increased cellular density, nuclear pleomorphism, blurring of the papillary pattern with poorly structured sheets of tumour cells, areas of necrosis and frequent mitoses, usually > 2.5 mitoses/mm^2^ in a minimum of 2.3 mm^2^ (equating to > 5 mitoses/10 HPF of 0.23 mm^2^). An intraventricular location is also a criterion. Additionally, TP53 mutation analysis and the methylation profile of the CPC are desirable. In select cases, the demonstration of hypoploidy by genome-wide chromosomal copy-number analysis may also be beneficial.

CPCs can usually be identified by histological and immunophenotypic analysis. Genome-wide chromosomal copy-number analysis reveals complex chromosomal alterations and characteristic hypoploidy [20], which may assist in differentiating CPCs from atypical CPPs. Methylome analysis has uncovered three clinically distinct subgroups of CPTs, with all CPCs clustering within one subgroup along with prognostically unfavourable grade 1 and 2 CPTs [40].

### Neuroimaging features

CPCs tend to show more irregular contours, and demonstrate heterogeneous appearance, and enhancement. Typical features include invasion and oedema of the adjacent parenchyma or metastatic dissemination in the subarachnoid space or within the third, fourth or lateral ventricles. Moreover, cysts, calcifications and haemorrhage may be observed. Septation, ventricular entrapment or cysts can also occur within the ventricular system, possibly reflecting an inflammatory reaction to the tumour or related to tumoural haemorrhage [18].

On CT, CPCs are typically heterogeneous, appearing isointense to hyperdense compared to grey matter. Calcifications may be seen in 20–25% of cases. Contrast enhancement is usually prominent but heterogeneous, with areas indicating necrosis and evident cyst formation.

**Fig. 9:**
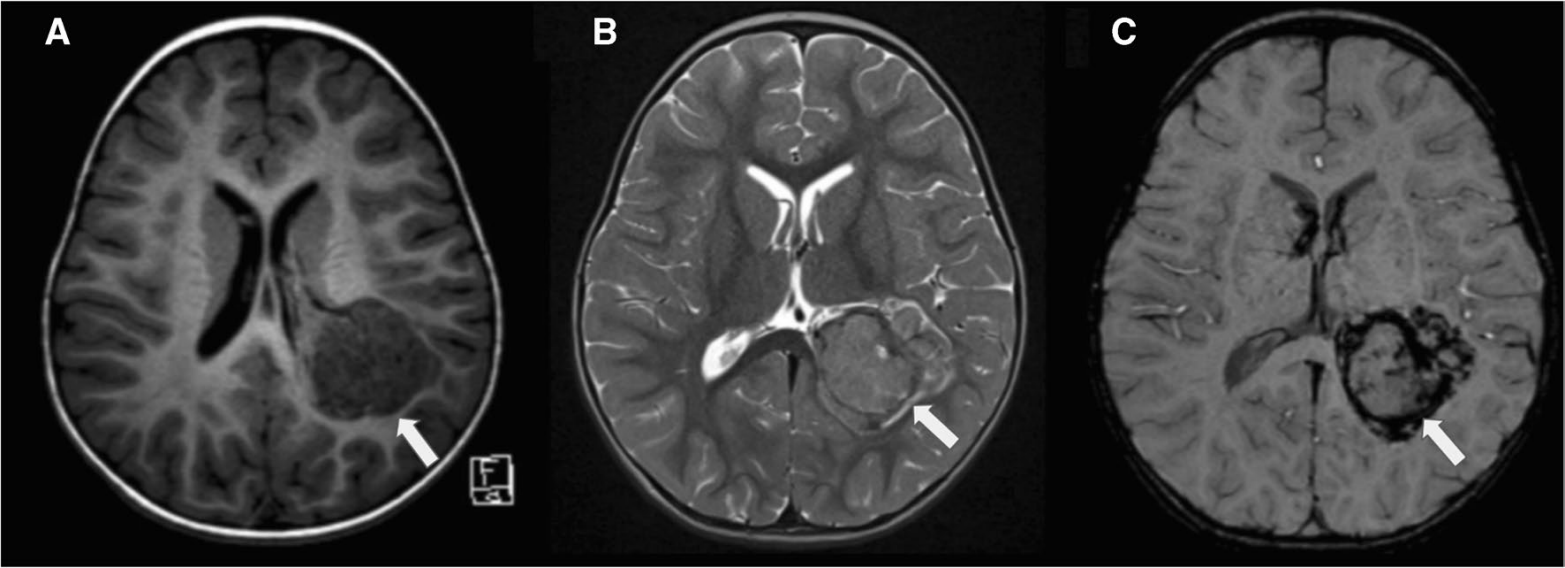
Choroid plexus carcinoma (whiter arrows) in a 24-month-old boy with headaches and recent seizures. **A** An axial T1-weighted image shows a macrolobulated, hypointense mass with signs of brain invasion and no hydrocephalus. **B** An axial T2-weighted image reveals that the lesion is predominantly isointense, with minimal surrounding CSF. In addition, there are signs of brain invasion and a mild degree of adjacent vasogenic edema. **C** An axial SWI image shows hypointense material, mainly at the periphery of the masses, consistent with haemorrhage

On MRI, CPCs typically appear as large, heterogeneous masses with ill-defined, lobulated and irregular margins. A sizable portion of the mass may not display a frond-like or cauliflower appearance. They primarily present isointense to hypointense signal relative to grey matter on T1WI, an isointense to hypointense signal with hyperintense cystic necrotic areas on T2WI, variable GRE or SWI signal from calcification/haemorrhage, and moderate to strong enhancement (Fig. 9). Septae or cysts may also occur within the ventricular system, possibly reflecting an inflammatory reaction to the tumour or related to tumoural haemorrhage (54). In addition, restricted diffusion may be seen. Given the potential for CSF dissemination, it is advisable to perform a post-contrast MRI of the spine to assess the extent of disease dissemination, which is observed in up to 44% of cases[41].

### Choroid plexus hyperplasia

### Background

CP hyperplasia, also known as benign villous hypertrophy or diffuse villous hyperplasia, is a rare condition characterised by an enlargement of the entire CP especially in both lateral ventricles. This condition occurs more commonly in young children but can be seen at any age. It is often associated with increased CSF production and nonobstructive hydrocephalus [42].

### Pathology

CP hyperplasia is characterised by an increased number of cuboidal to columnar epithelium cells with a hobnail appearance, resembling normal CP and without signs of nuclear crowding [43, 44]. The diagnosis of CP hyperplasia is typically made by excluding bilateral CP papillomas and demonstrating histological evidence of normal CP morphology [43].

### Neuroimaging features

Typical findings in CP hyperplasia are the diffuse enlargement of the CP, exhibiting homogeneous enhancement without discrete masses and possible association with communicating hydrocephalus [45, 46]. This condition may resemble the rare bilateral CPP; however, the latter typically presents as more focally nodular or lobular and is often asymmetrical. In such instances, imaging becomes crucial to assess the presence or absence of focal CP masses and to rule out hydrocephalus.

On CT scans, individuals with CP hyperplasia often present with diffusely enlarged and enhancing bilateral CP, which can occur with or without dilated ventricles. No discernible masses are typically present [43, 44].

**Fig. 10:**
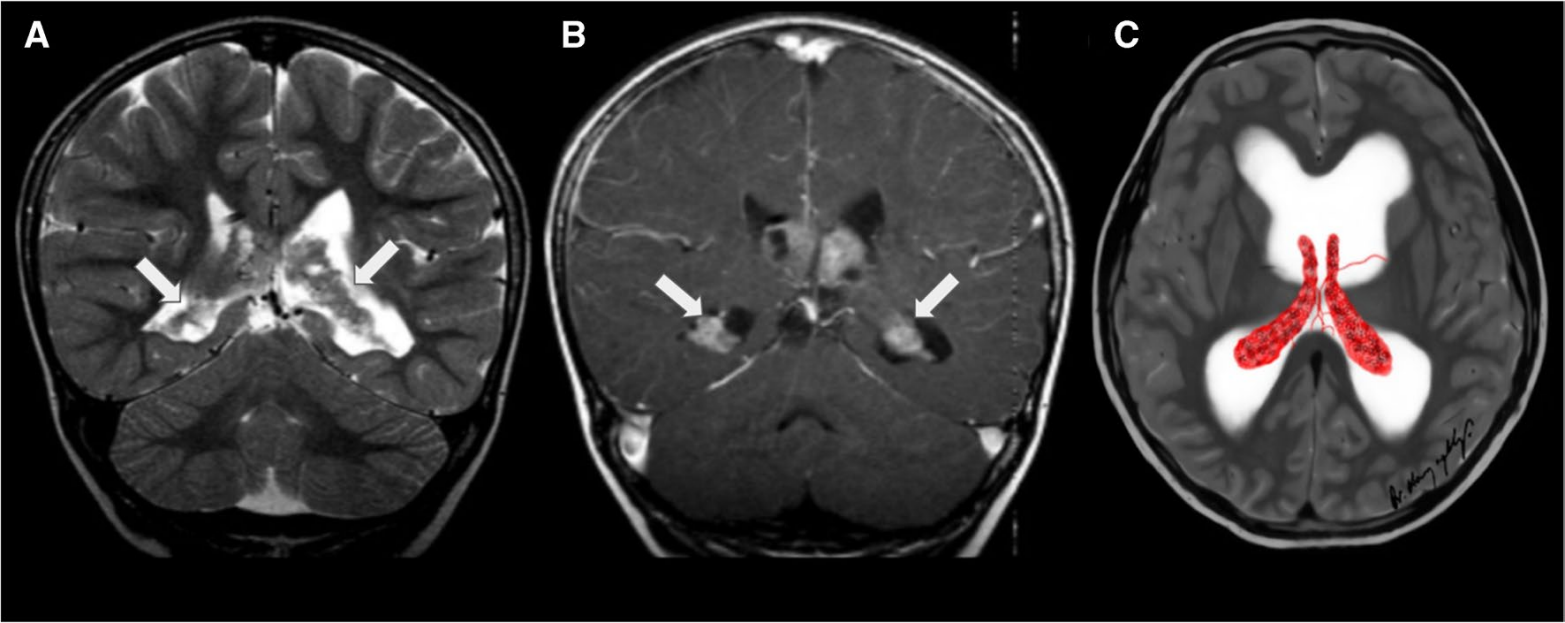
Choroid plexus hyperplasia (white arrows) in a 4-year-old girl with headaches due to hydrocephalus, treated with a ventricular drain. **A** A coronal T2-weighted image shows enlarged, hyperplastic, bilateral normal-appearing choroid plexus and mildly enlarged lateral ventricles. **B** A coronal contrast-enhanced T1-weighted image reveals that the choroid plexus demonstrates mild, non-nodular enhancement. **C** An illustration represents bilateral choroid plexus hyperplasia associated with moderate hydrocephalus

MRI, being superior to CT, can establish the diagnosis of CP hyperplasia by demonstrating a diffusely enlarged, homogeneously enhancing choroid plexus in a patient with or without communicating hydrocephalus [45]. Contrast-enhanced MRI often enables differentiation between the diffusely enlarged CP and the markedly enlarged, avidly enhancing, nodular, lobulated CPPs (Fig. 10) [43, 44].

## Meningioma

### Background

Meningiomas (WHO grades 1 to 3) are rare in children and are even less likely to occur in the ventricles [47]. While meningiomas can appear anywhere within the ventricular system [48], they are more commonly found in the atrium of the lateral ventricles [48, 49]. These tumours arise from arachnoid cells trapped within the CP [18] and exhibit no histological difference from the supratentorial meningiomas [50].

### Pathology

**Fig. 11:**
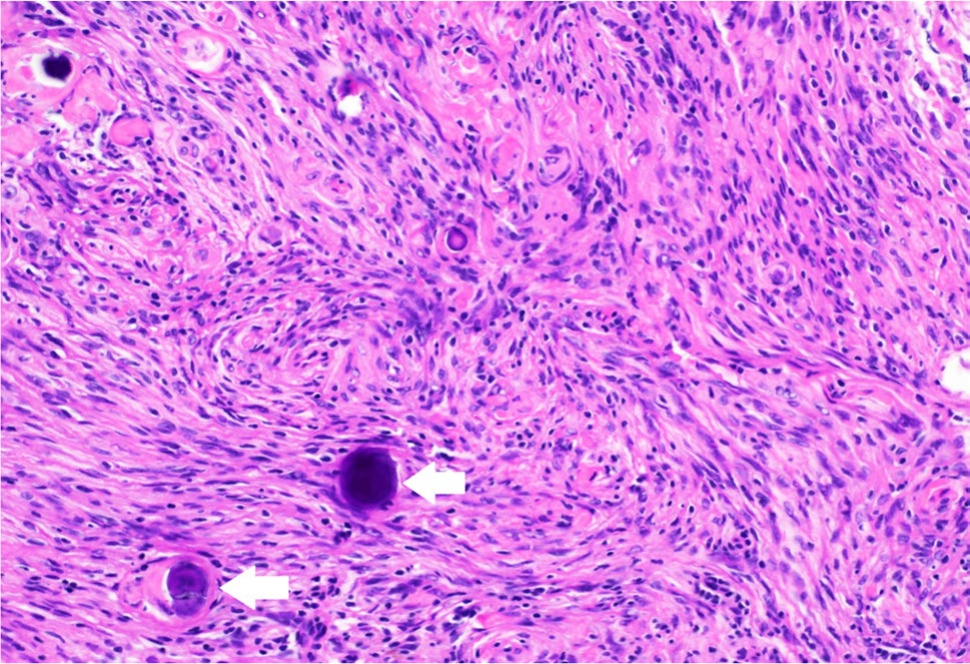
Meningioma—the image shows tumour cells with round to ovoid nuclei, growing in a lobulated to fascicular pattern, with intervening collagenous bands, focal whorls and calcifications (psammoma bodies, indicated by arrows). H&E stain, 20 × magnification

Intraventricular meningiomas can present with any histopathology type (predominantly fibrous, fibroblastic, meningothelial or psammomatous) as defined by WHO CNS5. Characteristically, meningiomas will display spherical formations of meningothelial cells, known as whorls, which may eventually mineralise into psammoma bodies (Fig. 11). Other common features include central chromatin clearing in the nucleus and the presence of intranuclear cytoplasmic pseudoinclusions [51].

According to WHO CNS5, the diagnostic criteria for meningioma typically require a combination of several elements. These include classic histopathological features corresponding to at least one meningioma subtype, suggestive histopathological features combined with biallelic inactivation of NF2 or other classic drivers of conventional meningioma (TRAF7, AKT1, KLF4, SMO, PIK3CA), clear cell meningioma (SMARCE1) or rhabdoid meningioma (BAP1). Alternatively, suggestive histopathological features combined with one of the defined DNA methylation classes of meningioma could be used for diagnosis. EMA immunoreactivity, strong and diffuse SSTR2A immunoreactivity and the presence of classic copy-number alterations of NF2-mutant meningioma, such as monosomy 22/22q in lower-grade meningiomas, with additional losses of 1p, 6, 10q, 14q and/or 18 in higher-grade meningiomas, are also desirable in the diagnostic process.

### Neuroimaging features

**Fig. 12:**
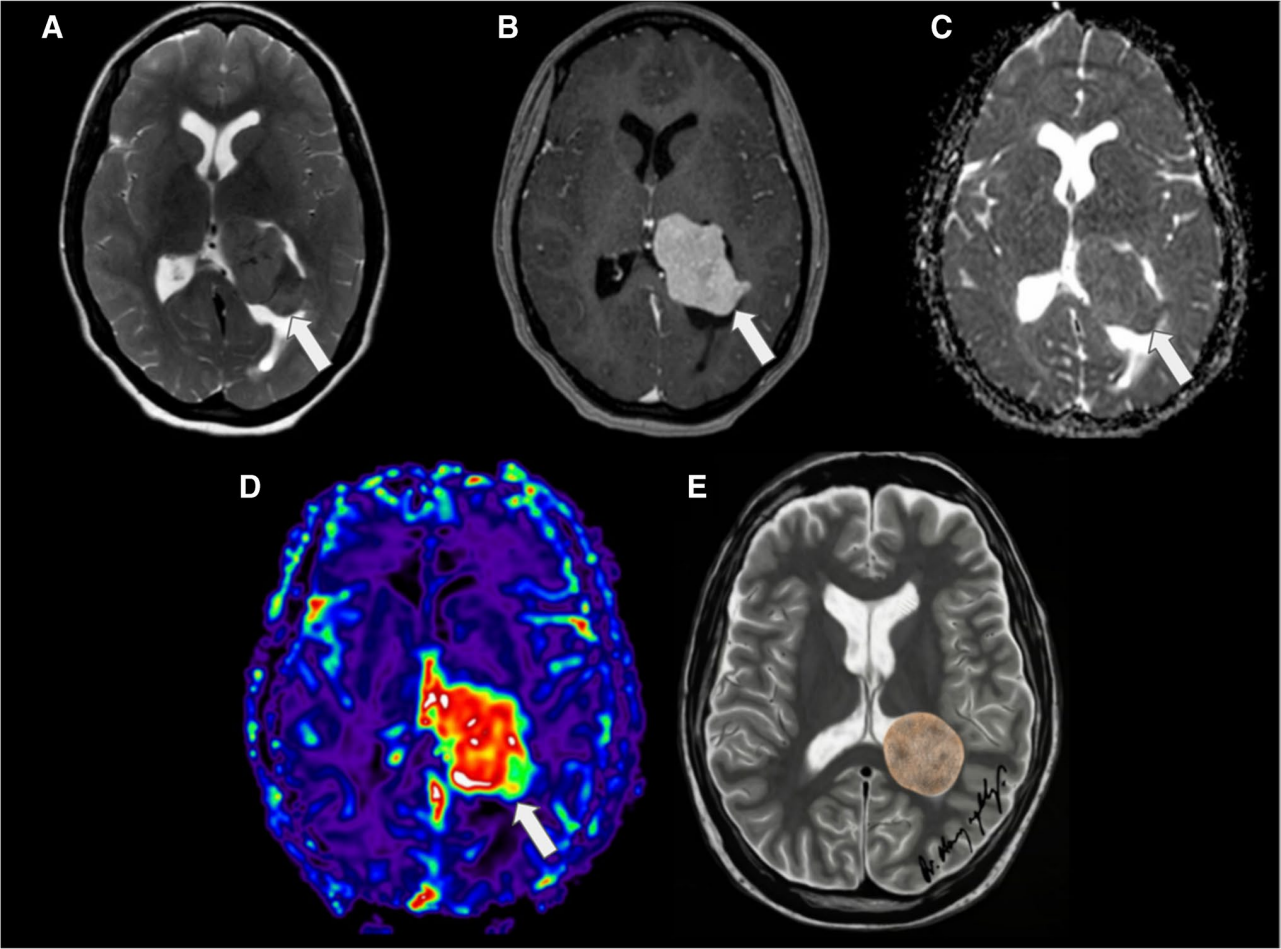
Intraventricular meningioma (white arrows) in a 15-year-old girl. **A** An axial T2-weighted image shows an isointense, lobulated, well-defined mass in the left lateral ventricle. **B** An axial contrast-enhanced T1-weighted image reveals that the lesion markedly and homogeneously enhances. **C** An axial apparent diffusion coefficient map indicates that the mass does not have restricted diffusion. **D** An axial arterial spin labelling perfusion image shows that the lesion has diffusely increased perfusion. **E** An illustration represents a typical case of a lateral ventricular meningioma with smooth contours and no signs of parenchymal invasion. Case courtesy of Dr. Guilherme Cassia, Brasília, Brazil

Meningiomas are typically well-defined lesions that display intense and relatively uniform enhancement. Occasionally, they may contain areas of central necrosis or calcification that do not enhance [52] (Fig. 12).

On CT, meningiomas appear as well-defined lobulated masses with moderate to high density and may also show calcification in up to 50% of cases.

**Fig. 13:**
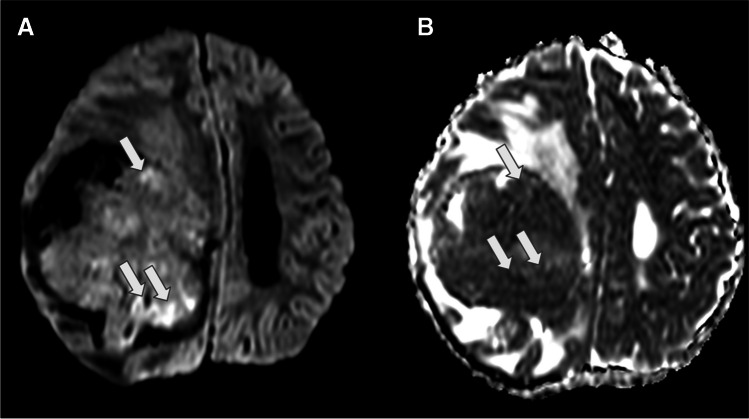
Intraventricular meningioma in the right lateral ventricle in a 9-year-old girl with minimal diffusion restriction. The large mass demonstrates a few areas of increased DWI signal (white arrows) in **A**, with corresponding low values on the ADC map (white arrows) in **B**

On MRI, meningiomas typically appear as a mass with an intermediate to hypointense signal on T1WI and an intermediate to hyperintense signal on T2WI, with intense solid enhancement often seen in highly vascular lesions [18]. Reduced diffusion (Fig. 13), seen as low values on the apparent diffusion coefficient maps, can be observed in some meningiomas, which might reflect high cellular density (Fig. 12) [53]. MRS may show a high choline peak and the presence of an alanine doublet at 1.48 ppm. The peaks for N-acetyl-aspartate and creatine are usually reduced or absent [53, 54]. Uncorrected perfusion-weighted images may reveal high blood volume and cerebral blood flow in some tumours, possibly due to immature and tortuous tumour vessels, resulting in increased leakage of contrast agents from vessels into the extravascular space [55, 56].

## Intraventricular arteriovenous malformations

### Background

CNS arteriovenous malformations (AVM) are generally considered to be congenital vascular malformations, originating during early foetal stages when newly formed blood vessels begin to differentiate into primitive arteries, capillaries and veins [57]. AVMs represent an abnormal shunt connection between arteries and veins, composed of feeding arteries, draining veins and a tangle of vessels known as the nidus, which is situated between the feeding arteries and draining veins and lacks a capillary bed [58]. AVMs can be classified as *diffuse* when neural tissue is interposed within the abnormal vessels, or *glomerular* when there is no brain tissue within the nidus [59]. AVMs are found throughout the CNS, with a higher prevalence in the supratentorial compartment [60]. Intraventricular AVMs account for approximately 4% of all AVMs in children, most commonly located in the lateral ventricles [61].

### Pathology

Microscopically, AVMs present numerous arteries and veins of varying sizes. They can also exhibit various blood clots and hemosiderin deposits due to their propensity to bleed [61]. Brain tissue can be observed between the abnormal vessels.

### Neuroimaging features

Brain digital subtraction angiography is the gold standard for evaluating and diagnosing AVMs [62]. It excels at delineating the arterial supply, location and number of feeding vessels, the nidus and the venous drainage of an AVM and demonstrates shunting (early venous drainage). Alternatively, intraventricular AVMs can be diagnosed using CT and MRI. In both CT and MRI, the lesion will enhance with contrast [61]. CT and MRI can also aid in grading AVMs based on the Spetzler-Martin AVM grading system, which assigns points for various imaging features of intracranial AVMs. Points are awarded based on the size of AVMs, involvement of the eloquent brain and pattern of venous drainage. The system final score helps predict the risk of morbidity/mortality from surgery [63].

CT is often used when intraventricular AVMs present with acute intraventricular haemorrhage. In such cases, there will be high-density intraventricular material due to the haemorrhage with or without a fluid–fluid level [57]. In the absence of haemorrhage, AVMs show intermediate to high attenuation and very intense enhancement after contrast administration [61]. On CT angiography, AVMs typically appear as a tangle of contrast-enhancing vessels [64]. Occasionally, scattered calcifications may also be observed.

**Fig. 14:**
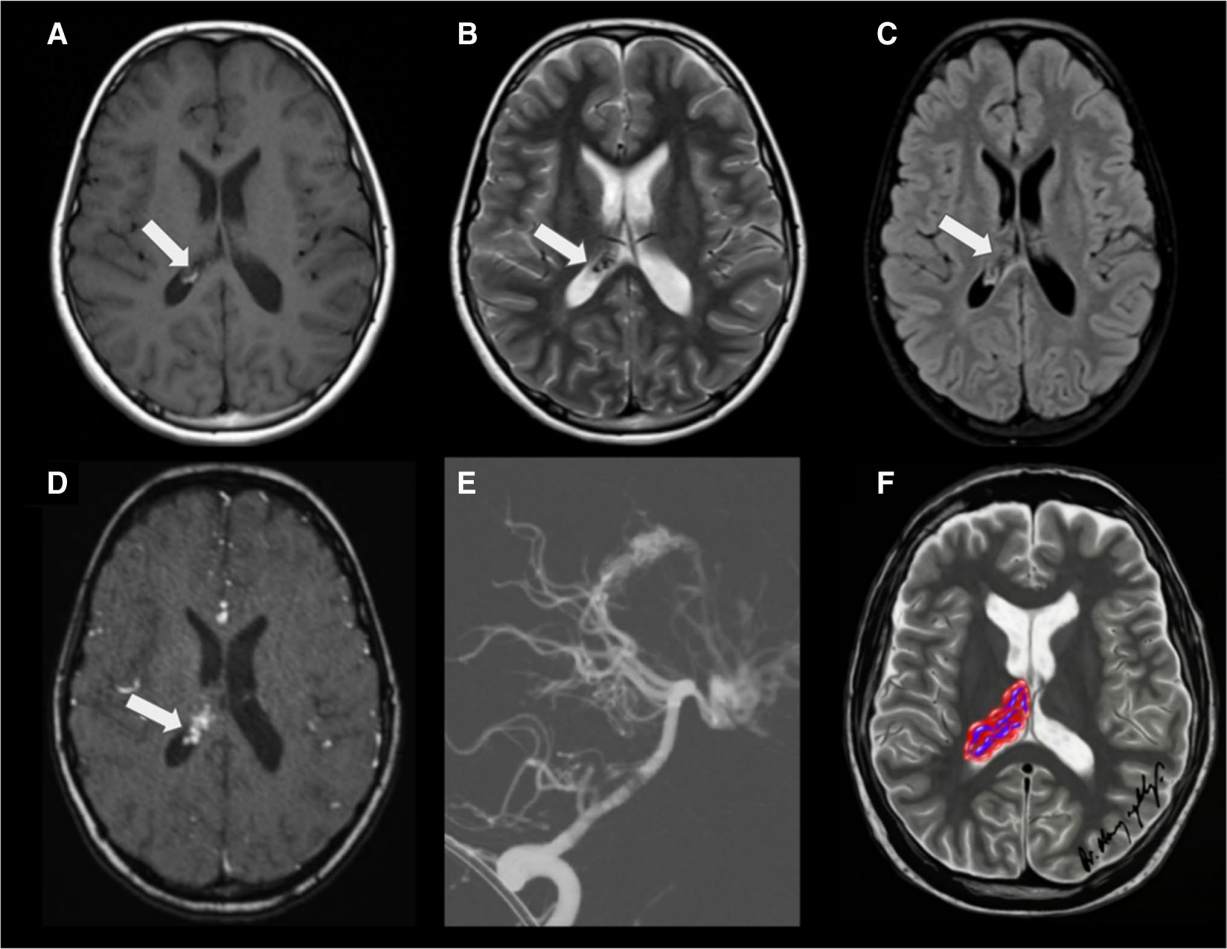
Intraventricular haemorrhagic arteriovenous malformation (white arrows) in a 10-year-old girl. **A** An axial T1-weighted image shows a slightly and asymmetrically enlarged choroid plexus in the right lateral ventricle, with a small focus of hyperintense signal due to recent bleeding. **B** An axial T2-weighted image demonstrates multiple small flow voids, in keeping with the presence of tortuous vessels within the enlarged choroid plexus. **C** An axial FLAIR image reveals that the choroid plexus is enlarged and associated with mild prominence of the lateral ventricle. **D** An axial source image from a time-of-flight MRA displays marked vascular dilatation and tortuosity within the choroid plexus. **E** A conventional catheter angiogram with vertebral artery injection shows significant dilation of branches of the lateral posterior choroidal artery, which feed the arteriovenous malformation. **F** An illustration representing a case of arteriovenous malformation in the right lateral ventricle

On MRI, AVMs typically appear as markedly hypointense lesions on both T1WI and T2WI, which represent flow voids due to fast flow and they intensely enhance following contrast administration [61]. Classically, AVMs are visible on T2WI images as a tangle of signal voids where large feeding arteries and drains can be easily identified. Blood-sensitive sequences, including gradient-recalled echo, T2-weighted and susceptibility-weighted imaging, can detect the presence of hemosiderin, an indicator of prior haemorrhage or calcification (Fig. 14) [64]. Large arteriovenous malformations and dural arteriovenous fistulas can be easily diagnosed with routine anatomic MR imaging. However, identifying small lesions or monitoring lesion evolution following embolisation therapies can be highly challenging. In fact, it has been proposed that arterial spin labelling (ASL) demonstrated strong performance for the detection compared to conventional CT angiography. Nevertheless, there are some concerns about the use of ASL in lesions with slow or fast shunting, as the venous ASL signal might be suboptimal or missed [65, 66].

## Choroid plexus lipoma

### Background

Intracranial lipomas are rare congenital malformations, making up less than 0.1% of all intracranial tumours. Isolated CP lipomas are most frequently found in the trigone of the lateral ventricle. These lipomas originate from abnormal differentiation of the primitive meninges during the development of subarachnoid cisterns and undifferentiated mesenchyme around the brain [16]. While isolated CP lipomas are typically incidental and lack associated malformations, they can be related to or extend from pericallosal lipomas and may also be accompanied by dysgenesis of the corpus callosum [67].

### Pathology

Generally, CP lipomas are composed of mature fat cells, ensconced within connective tissue and blood vessels. However, due to their asymptomatic nature, literature discussing their histology is limited. These lipomas typically do not require surgical excision, primarily because of their significant vascularity and strong adhesion to the surrounding tissue, making the procedure potentially risky [68].

### Neuroimaging features

Given their congenital nature, CP lipomas may manifest as distinct hyperechoic masses on foetal ultrasound, typically located at the trigone of the lateral ventricle. Their lipid content allows for clear detection on CT and MRI scans, particularly with the use of fat saturation techniques. This can highlight the lipid components within the mass, eliminating the need for a histologic examination [16].

**Fig. 15:**
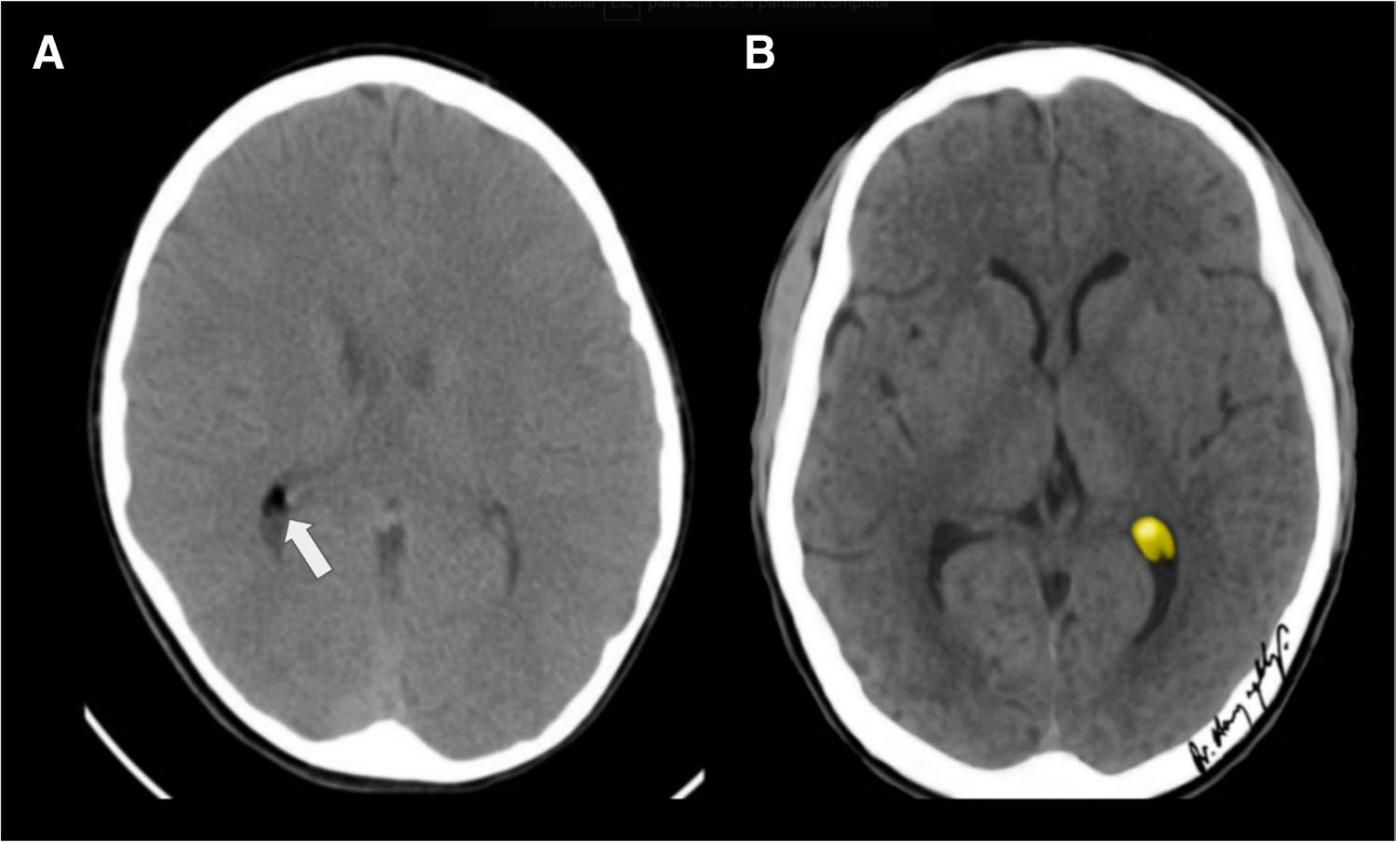
Choroid plexus lipoma (white arrow) incidentally found in a 10-year-old girl. **A** An axial non-enhanced contrast computed tomography scan shows a small lipid-containing nodule in the choroid plexus of the right lateral ventricle. **B** An illustration representing a choroid plexus lipoma

On CT scans, lipomas manifest as markedly hypodense regions that do not exhibit enhancement after contrast application (Fig. 15). Calcifications may sometimes be observed, either as curvilinear forms around the lipoma periphery or as nodular formations within the lesion centre [69]. Furthermore, they display a characteristic fat density on CT scans [16].

On MRI, lipomas present as hyperintense on T1WI, isointense to hyperintense on T2WI and hyperintense on FLAIR sequences. On fat-saturated sequences, the lipid signal within the masses is completely suppressed, appearing isointense to grey matter [69]. Occasionally, lipomas may mimic blood on SWI phase maps.

## Choroid plexus cyst

### Background

CP cysts develop when the neuroepithelium lining the interlobar clefts invaginates into the stroma, leading to the subsequent accumulation of CSF and debris. They occur throughout the ventricular system but are most frequently seen in the glomus of the lateral ventricles. CP cysts can be categorised into two types: antenatal and those that develop later in life. Antenatal CP cysts are observed in the foetus in approximately 1% of all pregnancies. Their prevalence is higher in foetuses with trisomy 18, trisomy 21 and Aicardi’s syndrome [16].

### Neuroimaging features

On prenatal ultrasound, antenatal CP cysts may appear as multiple cysts of variable sizes and sometimes as cysts with double walls. On CT and MRI, CP cysts typically display CSF signal characteristics.

On CT, CP cysts typically exhibit CSF density. However, they can occasionally appear hyperdense relative to the CSF. Additionally, the rims of these cysts may contain calcifications [70].

**Fig. 16:**
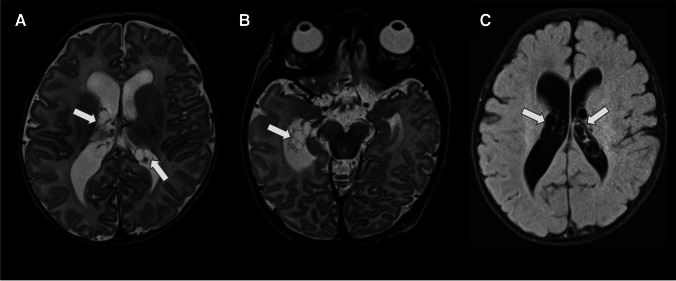
Choroid plexus cysts (white arrows) in a newborn. **A**, **B** Axial T2-weighted images display multiple small cysts in both lateral ventricles. **C** The axial FLAIR image demonstrates that the cysts have a signal similar to that of the CSF

On T1WI and T2WI MRI images, CP cysts usually display signal characteristics similar to those of CSF, making them difficult to identify (Fig. 16). Post-contrast imaging may reveal a sharply defined rim of peripheral enhancement, especially in adults. On FLAIR images, the contents of the cysts may appear hyperintense compared to the CSF.

## Choroid plexus xanthogranulomas

### Background

Xanthogranulomas, also known as xanthomas (not to be confused with juvenile xanthogranulomas, a distinct histiocytic process), or cholesterol granulomas, are benign lesions. They are characterised by cholesterol clefts, hemosiderin deposits, lymphocytic infiltration and fibrous proliferation. Intracranially, these are most commonly found in the lateral and third ventricles of the CP. CP xanthogranulomas are typically idiopathic, with an autopsy incidence ranging approximately between 1.6 and 7.0% [71].

### Pathology

Grossly, xanthogranulomas present as pale-yellow, soft, encapsulated lesions. Under microscopic examination, they exhibit foamy histiocytes surrounded by needle-like cholesterol crystal clefts and mononuclear inflammatory cells. Other features that may be observed include intralesional bleeding, fibrosis and focal calcifications [71, 72].

### Neuroimaging features

Xanthogranulomas are often bilateral, well-circumscribed, lobulated lesions in the lateral ventricles, frequently accompanied by a rim of calcification. The definitive diagnosis is established through histological examination, although this is often unnecessary as the majority of these lesions are asymptomatic [72].

On CT imaging, the appearance of xanthogranulomas can vary, ranging from hypodense to hyperdense compared to brain tissue. Generally, they present as spherical or ovoid lesions and may display a scattered rim of calcification [73].

**Fig. 17:**
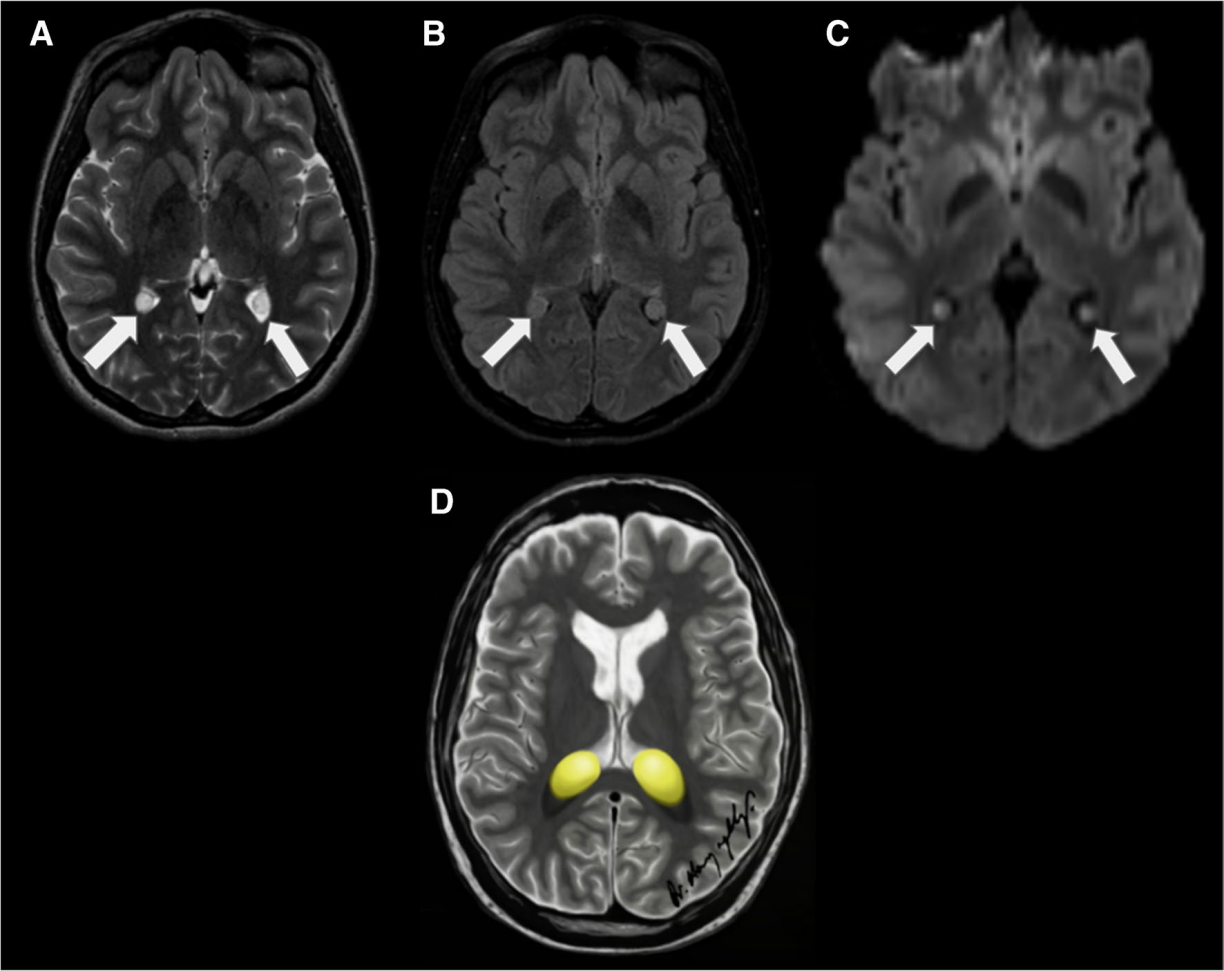
Bilateral choroid plexus xanthogranulomas (white arrows) in a 15-year-old male. **A** Axial T2-weighted image displays bilateral cystic lesions in the atria of the lateral ventricles. **B** These lesions are hyperintense relative to the CSF in the FLAIR image. **C** They are also hyperintense in the DWI. **D** An illustration representing bilateral choroid plexus xanthogranulomas

On MRI, xanthogranulomas typically present isointense or hypointensity on T1WI and hypointensity on T2WI. Their enhancement with gadolinium contrast is variable. It is common that the internal signal does not suppress on FLAIR imaging. The radiological features of xanthogranulomas are somewhat variable due to the heterogeneity of their content. Calcifications, which are often present in these lesions, appear hypointense on T2WI. Restricted diffusion and/or T2 shine-through on DWI is commonly seen in these lesions (Fig. 17).

## Atypical teratoid rhabdoid tumours

### Background

ATRT, WHO grade 4, is a highly malignant embryonal tumour that can occur in any region of the CNS, although it is most frequently found in the posterior fossa. Notably, an intraventricular location is remarkably rare [74]. The tumour exhibits a significant tendency towards leptomeningeal dissemination in roughly 15–30% of cases and is more commonly seen in children under the age of two [75]. ATRTs can be classified into three distinct molecular subgroups, each with preferred locations within the brain. These subgroups have been identified as follows: ATRT–Myelocytomatosis Oncogene (MYC) primarily occurs in supratentorial areas; ATRT-Tyrosine (TYR) is typically found in infratentorial regions and ATRT–Sonic Hedgehog (SHH) can occur in both supratentorial and infratentorial regions, which is heterogeneous with regard to age, tumour location, clinical and molecular features. It can be subdivided into SHH-1A, SHH-1B and SHH-2. Data suggest that molecular subgrouping of ATRT–SHH has prognostic relevance and might aid in stratifying patients [76, 77].

### Pathology

**Fig. 18:**
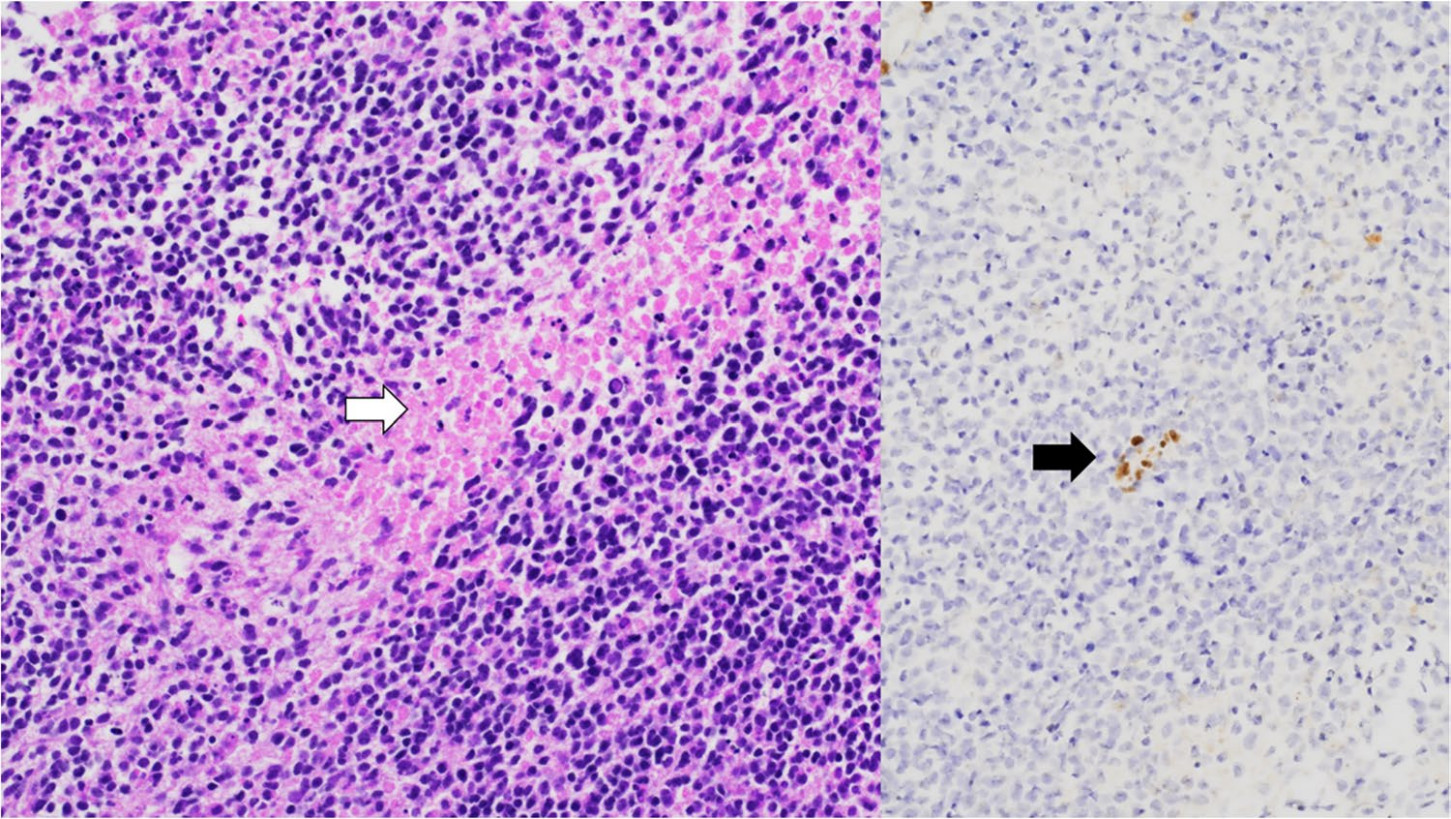
Atypical teratoid rhabdoid tumour—the left image depicts a primitive-appearing tumour with high mitotic activity and foci of necrosis (indicated by the arrow). This image is an H&E stain at 20 × magnification. The right image demonstrates a loss of INI-1 staining in the tumour nuclei, with retained staining in normal tissue (as seen in the vessel, indicated by the arrow). This image is an INI-1 immunostain at 20 × magnification

According to the WHO CNS5, the defining characteristic of ATRTs is the presence of an embryonal tumour with a polyimmunophenotype. On microscopy, ATRTs appear as clusters of highly atypical cells that may be scattered with various components of primitive neuroectodermal, mesenchymal and epithelial cells. Despite not consistently exhibiting the dominant morphology, rhabdoid cells display characteristics such as medium-sized, round to oval shapes with distinct borders, an eccentric nucleus and a prominent nucleolus. The tumours exhibit a diverse spectrum of immunoreactivity on immunohistochemical staining, with clusters of cells commonly positive for epithelial membrane antigen and vimentin [78]. The definitive diagnosis for ATRT relies on genetic/molecular analysis, specifically the loss of nuclear SMARCB1/INI1 or SMARCA4 expression in tumour cells. This is present in approximately 98% of cases (Fig. 18) [74, 75].

### Neuroimaging features

On imaging, ATRT will display heterogeneity, reflecting a combination of a tumour with solid and cystic components, with the presence of haemorrhage and necrosis [78].

On CT, ATRTs will appear hyperdense and show intense enhancement with contrast. Overall, they will display heterogeneously hyperdense due to the frequent presence of cystic and necrotic areas, calcifications and haemorrhage [78].

**Fig. 19:**
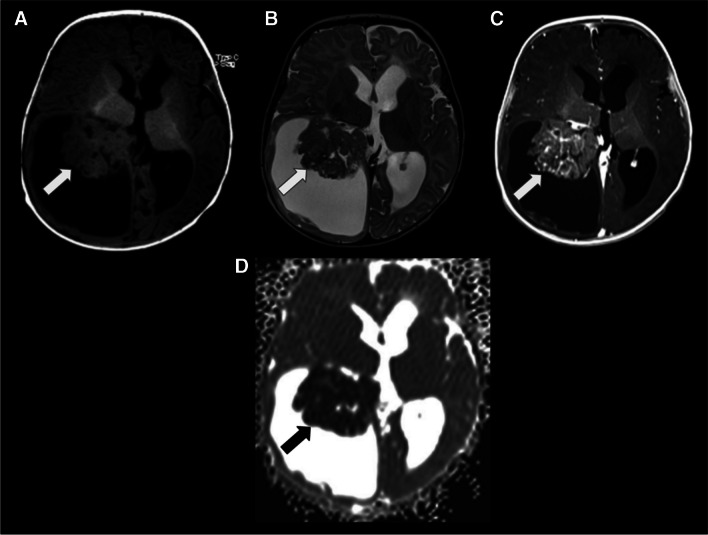
Intraventricular ATRT (whiter arrows). **A** The axial T1-weighted image displays an irregular, isointense, lobulated intraventricular mass associated with hydrocephalus. **B** The axial T2-weighted image reveals the mass to be diffusely hypointense, with central cystic/necrotic degeneration. Notably, the lesion is partially attached to the ventricular wall and associated with a marked enlargement of the right lateral occipital horn, likely entrapped. **C** The axial contrast-enhanced T1-weighted image indicates that the lesion has moderate and heterogeneous enhancement. **D** The axial apparent diffusion coefficient (ADC) map image demonstrates that the lesion exhibits marked restricted diffusion

On MRI, the solid component typically demonstrates isointense to hypointensity on T1WI, resembling grey matter. Due to their high cellularity, these tumours commonly exhibit increased signal intensity on DWI with low ADC values, which is also a characteristic shared with other embryonal tumours [74, 75] (Fig. 19). Leptomeningeal seeding is a relatively common occurrence. It has been reported in up to 30% of cases. Therefore, in situations where ATRT is suspected, it is recommended to conduct post-contrast imaging of the neuraxis to assess for the presence of leptomeningeal involvement. The detection of peripheral cysts and/or a bandlike “wavy” enhancement is important for distinguishing ATRT (across all molecular subgroups) from other tumour entities in children under the age of three [76].

## Choroid plexus metastases

### Background

Metastases to the CP from non-CNS tumours are extremely infrequent, representing 0.9–4.6% of all cerebral metastases. Neoplastic cells can access the CP through two distinct mechanisms: hematogenous or CSF seeding [79]. Among adults, renal and lung carcinomas are the primary sources giving rise to CP metastases. Metastatic spread predominantly occurs in the lateral ventricle, with the third and fourth ventricles being the sites most frequently involved [18]. In children, metastasis to the CP is even more infrequent with neuroblastoma, Wilms tumour and retinoblastoma being the most prevalent sources [80, 81]. However, intraventricular metastatic dissemination of paediatric CNS tumours is common and sporadically affects the CP. This phenomenon is predominantly observed in medulloblastoma, embryonal tumours, ependymomas and high-grade gliomas.

### Pathology

The gross pathology and histology of CP metastasis can exhibit variations based on the primary neoplasm [82].

### Neuroimaging features

The imaging pattern of CP metastasis demonstrates variations based on the specific features of the primary tumour [16]. CP metastasis can present as either a single or multiple lesion(s), with a notable tendency for concurrent multiple additional intracranial metastases [83]. Other observed features include marked enhancement, hydrocephalus, haemorrhage, oedema and invasion of the brain parenchyma [18, 80]. Solitary metastasis to the CP may mimic other CPs tumours, such as a meningioma or a primary CPT. Hence, a history of primary neoplasm should heighten the clinical suspicion for potential metastasis [18].

On CT scans, metastasis can demonstrate variable density influenced by factors such as cellularity and the presence of haemorrhage. Typically, pronounced enhancement is observed [84].

On MRI, the signal intensity of metastasis can exhibit variability. However, it generally demonstrates intense and homogeneous enhancement. Moreover, MRI provides a better depiction of oedema and invasion of adjacent brain structures [82].

## Conclusion

In conclusion, the evaluation of supratentorial intraventricular masses in children is a complex diagnostic challenge, compounded by the wide variety of potential lesions and their overlapping clinical and imaging features. This review underscores the importance of a systematic approach to differential diagnosis, necessitating consideration of not just the lesion’s appearance and location, but also the patient’s age and specific imaging characteristics.

Primary choroid plexus tumours, including choroid plexus papilloma, atypical choroid plexus papilloma and choroid plexus carcinoma, represent the most prevalent and clinically significant primary supratentorial intraventricular masses in this age group. A choroid plexus papilloma typically presents with a frond-like or cauliflower appearance, surrounded by cerebrospinal fluid, and shows homogeneous and intense enhancement. However, the absence of these characteristic imaging features is observed in atypical presentations of choroid plexus papillomas and other masses, necessitating careful consideration of alternative diagnoses.

The strategies outlined in this review offer a structured method for narrowing the differential diagnosis, aiding in the more accurate and timely identification of these lesions, which is crucial for guiding effective treatment planning and improving patient outcomes. Surgery remains the cornerstone of treatment for specific supratentorial intraventricular masses in children, making thorough preoperative evaluation essential for surgical planning and staging. This comprehensive approach allows for a comprehensive assessment of these tumour types, leading to a more precise and focused differential diagnosis.

These insights lay the groundwork for part 2 of this series, where we will delve into other specific lesion types, further enhancing our understanding of these complex intraventricular masses in paediatric patients.

## Data Availability

Yes.

## Code availability

NA.
